# Production Task Queue Optimization Based on Multi-Attribute Evaluation for Complex Product Assembly Workshop

**DOI:** 10.1371/journal.pone.0134343

**Published:** 2015-09-28

**Authors:** Lian-hui Li, Rong Mo

**Affiliations:** Ministry of Education Key Laboratory of Contemporary Design and Integrated Manufacturing Technology, Northwestern Polytechnical University, Xi’an 710072, Shaanxi, China; Southwest University, CHINA

## Abstract

The production task queue has a great significance for manufacturing resource allocation and scheduling decision. Man-made qualitative queue optimization method has a poor effect and makes the application difficult. A production task queue optimization method is proposed based on multi-attribute evaluation. According to the task attributes, the hierarchical multi-attribute model is established and the indicator quantization methods are given. To calculate the objective indicator weight, criteria importance through intercriteria correlation (CRITIC) is selected from three usual methods. To calculate the subjective indicator weight, BP neural network is used to determine the judge importance degree, and then the trapezoid fuzzy scale-rough AHP considering the judge importance degree is put forward. The balanced weight, which integrates the objective weight and the subjective weight, is calculated base on multi-weight contribution balance model. The technique for order preference by similarity to an ideal solution (TOPSIS) improved by replacing Euclidean distance with relative entropy distance is used to sequence the tasks and optimize the queue by the weighted indicator value. A case study is given to illustrate its correctness and feasibility.

## Introduction

Under the fierce market competition background, how to achieve the aim of just-in-time production is always a key factor of an enterprise’s survival and development. As one of the final and the most important phases of product manufacturing, assembly has a significant impact on the realization of just-in-time production [1–9]. The production task queue, which is formed by prioritizing the production tasks, is determined by the task attributes. In the complex product assembly process, the working time is long and production resources are limited. In the task queue, the manufacturing resource allocation should be inclined to the high priority task, and the scheduling decision should also pay more attention to the high priority task. If the low priority task is executed earlier than the high one, the whole production process will be delayed. Thus the production task queue should be optimized in a scientific and reasonable way.

For the complex product assembly workshop, manufacturing resource allocation and scheduling decision is very conducive to the realization of just-in-time production. Optimizing the task queue in a scientific and reasonable way can provide the basis for manufacturing resource allocation and scheduling decision. The related researches are mainly focused on scheduling algorithms [10–13] and assembly line balancing problem (ALBP) [14–17]. However, different tasks have different priorities. On the one hand, this influence mostly hasn’t been considered in the scheduling algorithms and ALBP [10–17]. On the other hand, ALBP is mainly applied in the batch production line. The complex product assembly has the characteristics of single or small batch production, discrete process, manual operation etc. The researches of ALBP are unsuitable for complex product assembly workshop.

In fact, production task queue optimization based on task attributes can be abstracted as a multi-attribute evaluation problem. The methods commonly used to solve this problem include analytic network process (ANP) [18], principal component analysis (PCA) [19], analytic hierarchy process (AHP) [20], maximum entropy method [21], fuzzy evaluation method [22], technique for order preference by similarity to ideal solution (TOPSIS) [23] etc. The individual application has a certain shortage, so the combination of these methods has been researched. Wang et al. proposed the method of multi-process plan evaluation base on fuzzy comprehensive evaluation and grey relational analysis [24]. Lou et al. proposed comprehensive evaluation model for water-saving development level of irrigation management in Sichuan province [25]. Yang et al. established the improved TOPSIS model in the comprehensive evaluation of groundwater quality [26].

From the related researches [18–26], the solutions of multi-attribute evaluation problem can be summarized as follows: firstly build the evaluation indicator system based on the object attributes, secondly calculate the indicator weight, and lastly sequence the objects by their weighted indicator values.

### How to Calculate the Indicator Weight

Based on the related literatures, the indicator weight calculating methods can be classified into three categories: objective weighting, subjectively weighting and combined weighting.

In objective weighting, entropy method [27], standard deviation method [28] and criteria importance through intercriteria correlation (CRITIC) [29] are widely applied.

In subjectively weighting, the exact number scale of 1~9 levels, which is used to express the subjective judgment in AHP [20], cannot completely reflect the judgment ambiguity. To represent the human-mind ambiguity, using the fuzzy comment such as ‘a little’ and ‘clear’ is more reasonable. Rough Sets (RS) is a mathematical tool to quantitatively analyze and process the imprecise, inconsistent and incomplete information [30]. The true perception of the judges can be apperceived more deeply with RS method. So it is widely applied in fault diagnosis [31–32], prediction and control [33–34], pattern recognition [35–36] and data mining [37–39] etc. If the indicator system has a large number of attributes and complex relationships, using RS method to determine the weight is often with a heavy calculation workload and low feasibility. Thus, Wang et al. defined the concept of rough number and rough boundary interval and proposed a rough AHP method [40]. In the group decision, the experience and wisdom of every judge should be fully exploited. Because the judges have different experiences, abilities and employment positions, they affect the result of group decision in a different degree. It is not reasonable that different judges are regarded as equal in subjectively weighting [24,26,31].

In combined weighting, both the objective indicator value differences and the subjective judge preferences are considered to determine the indicator weight. So the combined weighting can reduce the information loss caused by the individual objective or subjective weighting. Some methods of combined weighting have been put forward: multiplying combination [26], experience factor combination [41] and optimized combination [24,42]. If the objective weight vector is equal to the subjective weight vector, the combined weight vector will be equal to the either one. If the subjective weight value of an indicator is unequal to its objective weight value, the combined weight value will be in between. In the methods of multiplying combination [26] and optimization combination [24,42], the above two points cannot be guaranteed. In the method of experience factor combination [41], the dependence on the experience factor will increase the influence of the subjective preference.

### How to Sequence the Objects

TOPSIS is a classic sequencing method for multi-attribute evaluation. Euclidean distances between the evaluation object and the two ideal points are used to calculate the closeness [23]. The objects on the perpendicular bisector of two ideal points have the same closeness and cannot be distinguished [43,44]. So some improved methods have been proposed such as the improved TOPSIS by angle measure evaluation [43] and the improved TOPSIS by vertical projection [44]. In the improved TOPSIS by angle measure evaluation [43], the angle closeness between the evaluation object and the ideal point is considered but the length difference is ignored. If two objects have the same angle closeness and different length, the sequencing result will be wrong. In the improved TOPSIS by vertical projection [44], if two or more objects have the same projective point on the connection line of two ideal points, they will not be distinguished.

## Methods

**Fig 1:**
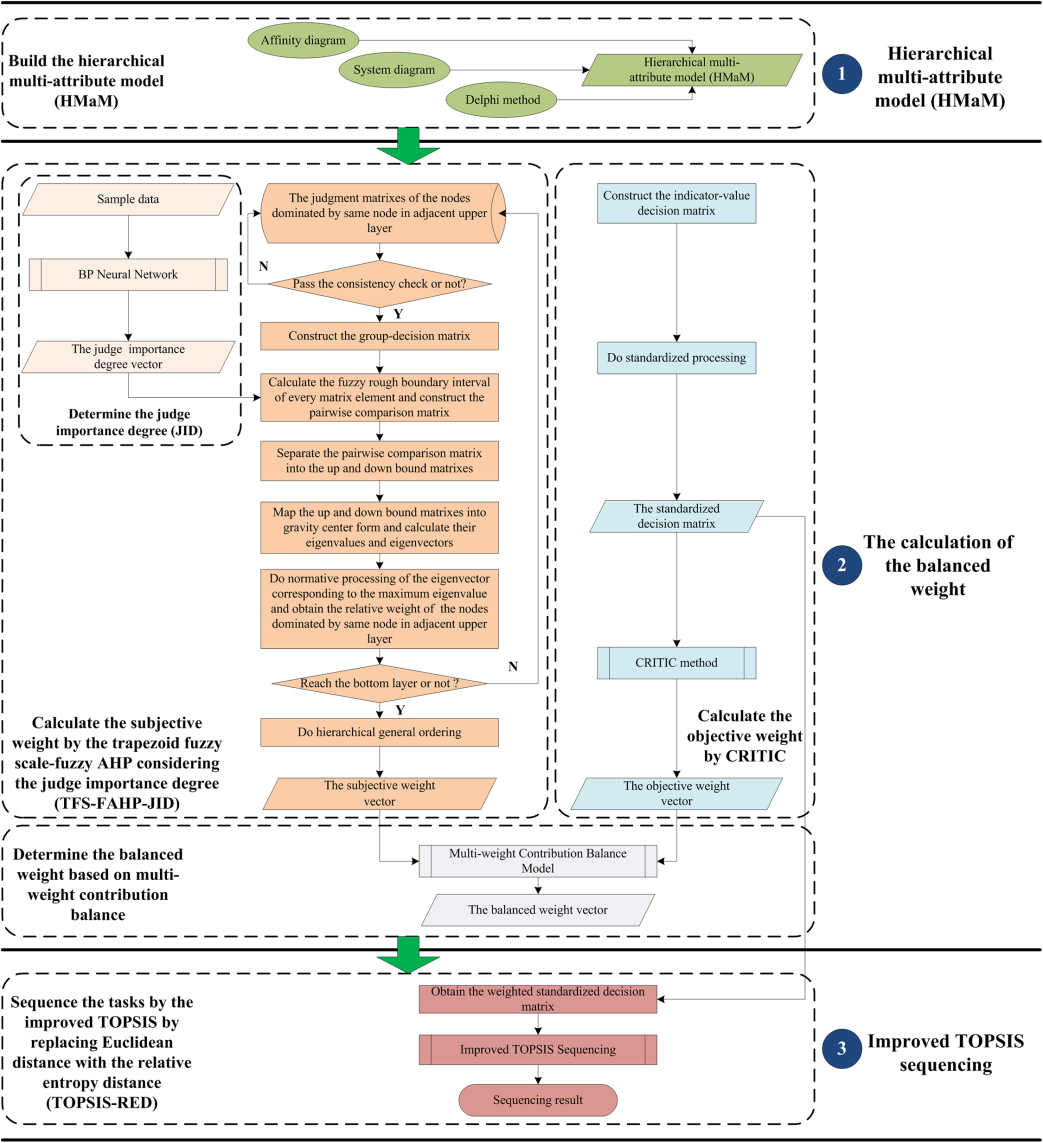
The flowchart of the production task queue optimization method.

The production task queue has a great significance for manufacturing resource allocation and scheduling decision. To solve the problem of poor effect and application difficulty of the man-made qualitative queue optimization, we propose a production task queue optimization method based on multi-attribute evaluation as shown in Fig 1. Firstly, the hierarchical multi-attribute model (HMaM) of the objects is built. Secondly, the indicator weight vector is calculated based on the multi-weight contribution balance. Lastly, the improved TOPSIS by replacing Euclidean distance with the relative entropy distance is proposed to sequence the tasks by the weighted indicator values.

### Hierarchical Multi-attribute Model

The methods of affinity diagram [45], system diagram [46,47] and Delphi method [48] are used to make the task attributes hierarchical. After some proper simplification, HMaM is built in Fig 1.

**Fig 2:**
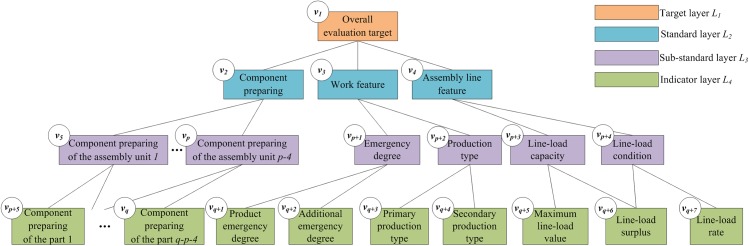
The structure of HMaM.

As is shown in Fig 2, HMaM includes four layers: the target layer *L*_1_, the standard layer *L*_2_, the sub-standard layer *L*_3_ and the indicator layer *L*_4_. The formal description of HMaM is *M =* {*V*, *E*} as follows:

1. *V* = {*v*_1_, *v*_2_, …, *v*_*n*_} represents the node set. *v*_1∈_ *L*_1_, *v*_2_,*v*_3_,*v*_4∈_ *L*_2_, *v*_5_,*v*_6_,…,*v*_*p*+4∈_ *L*_3_, *v*_*p*+5_, *v*_*p*+6_,…,*v*_q∈_ *L*_4_. *L*_1_, *L*_2_, *L*_3_, *L*_4_ ⊂ *V*.
2. *E* = {$\left(v_{i},v_{j},\delta_{j}^{i}\right)$| *v*_*i*_,*v*_j∈_ *V*, 0<$\delta_{j}^{i}$≤1} is the relationship set. $\left(v_{i},v_{j},\delta_{j}^{i}\right)$ represents the relationship between *v*_*i*_ and *v*_*j*_, and $\delta_{j}^{i}$ represents the relative weight of *v*_*j*_ dominated by *v*_*i*_. $\forall (v_{i},v_{j},\delta_{j}^{i})\in E,\;v_{i}\in L_{k}\vee v_{j}\in L_{k+1}(k=1,2,3)$.

As shown in Fig 2, the overall evaluation target *v*_1_ is divided into three standards, namely component preparing standard *v*_2_, work feature standard *v*_3_ and assembly line feature standard *v*_4_. The description and quantization of the indicators are as follows:

1. Component preparing standard *v*_2_
  In the assembly process, the complex product is generally divided into several assembly units, and each assembly unit is composed of several parts. For example, the aero-engine is divided into rotor unit, combustion unit, transmission unit etc. Rotor unit is composed of turbine part, combustion unit is composed of combustor part, and transmission unit is composed of decelerator part and transmission casing part. So the standard *v*_2_ can be divided into the sub-standards *v*_5_, *v*_6_,…, *v*_*p*_ which mean the component preparing status of assembly units. The sub-standard *v*_*i*_(5*≤i≤p*) can be divided into the indicators *v*_*p*+5_,*v*_*p*+6_,…, *v*_*q*_ which mean the component preparing status of parts.
2. Work feature Standard *v*_3_
  The standard *v*_3_ can be divided into the sub-standards *v*_*p*+1_ (emergency degree) and *v*_*p*+2_ (production type).

The sub-standard *v*_*p*+1_ is composed of the indicators *v*_q+1_ (product emergency degree) and *v*_q+2_ (additional emergency degree). The indicator *v*_q+1_ means the product emergency degree specified in production plan. The indicator *v*_q+2_ means the task emergency degree attached by the assembly workshop managers.

The sub-standard *v*_*p*+2_ is composed of the indicators *v*_*q+3*_ (primary production type) and *v*_q+4_ (secondary production type). The indicator *v*_q+3_ means the production types such as development production, small batch production, maintenance, repair, overhaul etc. The indicator *v*_q+4_ means the customer types of the product corresponding to the task.

1. Assembly line feature standard *v*_*4*_
  The standard *v*_*4*_ means the features of the assembly line which the task will be assigned to. The standard *v*_*4*_ can be divided into the sub-standards *v*_*p*+3_ (line-load capacity) and *v*_*p*+4_ (line-load condition).

The sub-standard *v*_*p*+3_ is composed of the indicators *v*_q+5_ (maximum line-load value) and *v*_q+6_ (line-load surplus). The indicator *v*_q+5_ means the maximum number that the assembly line can undertake tasks simultaneously. The indicator *v*_q+6_ is the difference between the tasks being undertaken and the maximum line-load.

The sub-standard *v*_*p*+4_ is composed of the indicators *v*_q+6_ (line-load surplus) and *v*_q+7_ (line-load rate). The indicator *v*_q+7_ is the ratio of the tasks being undertaken to the maximum line-load. If the line-load rate *v*_q+7_ is high, the assembly line is busy and the ability to accept new tasks is low.

The quantization methods of the indicators are shown in Table 1.

**Table 1:** The quantization methods of the indicators.

Indicator	Quantization method	Remarks
*v*_*p*+5_ (component preparing status of the part **1**)	*v*_*p*+5_ = *F*_p+5_/*C*_p+5_	*C*_p+5_ means the total component number of the part **1** and *F*_p+5_ means the prepared component number of the part **1**.
*v*_*p*+6_(component preparing status of the part **2**)	*v*_*p*+6_ = *F*_p+6_/*C*_p+6_	*C*_p+6_ means the total component number of the part **2** and *F*_p+6_ means the prepared component number of the part **2**.
*…*	*…*	*…*
*v*_*q*_ (component preparing status of the part ***q-p-*4**)	*v*_*q*_ = *F*_*q*_/*C*_*q*_	*C*_*q*_ means the total component number of the part ***q-p-*4** and *F*_*q*_ means the prepared component number of the part ***q-p-*4**.
*v*_q+1_ (product emergency degree)	*v*_q+1_ ∈ {0.8, 0.6, 0.4, 0.2}	Emergency degree is divided into four levels: especially emergency, more emergency, emergency and general, which correspond to the evaluation value 0.8, 0.6, 0.4 and 0.2.
*v*_q+2_ (additional emergency degree)	As same as *v*_q+1_.	As same as *v*_q+1_.
*v*_q+3_ (primary production type)	*v*_q+3_ = $\sum_{k=1}^{n}\varepsilon_{ik}\cdot \frac{k}{n+1}$.	Take ***e*** experts to evaluate ***p*** primary production types *PT*_1_,*PT*_2_, *…*,*PT*_*p*_ with fuzzy mathematic method (the fuzzy comment set is *Rem* = {*Rem*_1_,*Rem*_2_, …, *Rem*_*n*_} which correspond to the fuzzy evaluation value set *Val* = {1/(*n*+1),2/(*n*+1), …, *n*/(*n*+1)}). For *PT*_*i*_ ∈ *PT* (1≤*i*≤*p*), the fuzzy comment set of ***e*** experts is *PT_Rem* = {*PT_Rem*_*1*_,*PT_Rem*_*2*_,*…*, *PT_Rem*_*e*_}(*PT_Rem*_*j*_ ∈ *Rem*,1≤*j*≤*e*), and the membership grade of *Rem*_*k*_ ∈ *Rem* can be expressed as *ε*_*ik*_ = *e*_*ik*_/*e* where *e*_*ik*_ is the number of the fuzzy comment *Rem*_*k*_ in *PT_Rem*. So if the task’s primary production type (*v*_*q+3*_) is *PT*_*i*_, the indicator value is *v*_q+3_ = $\sum_{k=1}^{n}\varepsilon_{ik}\cdot \frac{k}{n+1}$.
*v*_q+4_ (secondary production type)	As same as *v*_q+3_.	As same as *v*_q+3_.
*v*_q+5_ (maximum line-load value)	*v*_q+5_ = *LN*_*i*_/max{*LN*_*i*_}	There are ***l*** tasks and ***W*** assembly lines (***l*** ≤***W***) and the tasks will be assigned to different assembly lines. *LN*_*i*_ is the maximum line-load of the line *i* and *r_LN*_*i*_ is the number of the tasks being undertaken by the line *i*, 1≤*i*≤*W*, 0≤*r_LN*_*i*_ <*LN*_*i*_.
*v*_q+6_ (line-load margin)	*v*_q+6_ = (*LN*_*i*_–*r_LN*_*i*_)/max{*LN*_*i*_–*r_LN*_*i*_}	As same as *v*_q+5_.
*v*_q+7_ (line-load rate)	*v*_q+7_ = (*LN*_*i*_–*r_LN*_*i*_)/*LN*_*i*_	As same as *v*_q+5_.

### Objective Weight Calculation

In this paper, there are *m* indicators (*v*_*p+5*_, *v*_*p+6*_,…, *v*_*q*_, *v*_q+1_, *v*_q+2_,…, *v*_q+7_, so *m* = *q-p*+3) and *l* evaluation objects. The indicator value decision matrix is defined as *T =* (*t*_*ij*_)_*l×m*_ in which *t*_*ij*_ represents the value of the object *i* on the indicator *v*_*j*_ (0≤*i≤l*, 0≤*j≤m*). To eliminate the dimension effects of different indicators, the standardized processing is carried on and the standardized decision matrix is *Z =* (*z*_*ij*_)_*l×m*_. For the efficiency type indicators (*v*_*p*+5_,*v*_*p*+6_,…,*v*_*q*_,*v*_q+1_,*v*_q+2_,…, *v*_q+7_), *z*_*ij*_ = *t*_*ij*_/*t*_max_(*j*). For the cost type indicators (*v*_*q+7*_), *z*_*ij*_ = *t*_min_(*j*)/*t*_*ij*_ where *t*_max_(*j*) = max(*t*_1*j*_,*t*_2*j*_,⋯,*t*_*lj*_),*t*_min_(*j*) = min(*t*_1*j*_,*t*_2*j*_,⋯,*t*_*lj*_).

The usual methods to calculate the objective weight are as follows:

1. Entropy method

For *m* indicators, the entropy value of the indicator *v*_*j*_ is:

$$EV_{j}=-\frac{1}{\ln m}\sum_{i=1}^{l}\frac{z_{ij}}{z_{\;,j}}\ln\frac{z_{ij}}{z_{\;,j}}\;(z_{\;,j}=\sum_{i=1}^{l}z_{ij})$$ (1)

The entropy value *EV*_*j*_ is smaller, the difference of the values of all objects on the indicator *v*_*j*_ is more obvious. So the indicator *v*_*j*_ is more important. Using entropy method, the objective weight of the indicator *v*_*j*_ is:

$$\chi_{v_{j}}=\left(1-EV_{j}\right)/\sum_{j=1}^{m}\left(1-EV_{j}\right)$$ (2)

2. Standard deviation method

Using standard deviation method, the objective weight of the indicator *v*_*j*_ is:

$$\chi_{v_{j}}=\sigma_{j}/\sum_{i=1}^{m}\sigma_{i}$$ (3)

where $\sigma_{j}=\sqrt{\frac{1}{l}\sum_{i=1}^{l}{\left(z_{ij}-\frac{\sum_{i=1}^{l}z_{ij}}{l}\right)}^{2}}$ is the standard deviation of the indicator *v*_*j*_.

3. CRITIC

The correlation coefficient between the indicator *v*_*i*_ and the indicator *v*_*j*_ is:

$$ccr_{ij}=\frac{\sum_{k=1}^{l}\left(z_{ki}-\bar{z_{,i}})(z_{kj}-\bar{z_{,j}}\right)}{\sqrt{\sum_{k=1}^{l}{\left(z_{ki}-\bar{z_{,i}}\right)}^{2}}\sqrt{\sum_{k=1}^{l}{\left(z_{kj}-\bar{z_{,j}}\right)}^{2}}}$$ (4)

where $\bar{y_{,i}},\;\bar{y_{,j}}$ are the arithmetic average value of *v*_*i*_ and *v*_*j*_.

The conflict degree between *v*_*j*_ and other indicators is:

$$R_{j}=\sum_{i=1}^{m}\left(1-ccr_{ij}\right)$$ (5)

Using CRITIC, the objective weight of the indicator *v*_*j*_ is:

$$\chi_{v_{j}}=C_{j}/\sum_{i=1}^{m}C_{i}$$ (6)

where *C*_*j*_ = *σ*_*j*_ *R*_*j*_ is the information volume of *v*_*j*_.

### Subjective Weight Calculation

#### BP Neural Network-based Judge Importance Degree Determination

Different judges have different experiences, abilities and employment positions, so their distinguishing importance degree should be taken into consideration. BP neural network has the ability of self-learning and makes the importance degree assignment more scientific and reasonable [49,50]. In this paper, we propose a BP neural network-based method to solve the judge importance degree (JID) determination problem in group decision.

In the JID assignment model base on BP neural network, several successful decisions including sequencing judgments and actual sorting result are necessary as the sample data. For a successful decision, the sequencing judgment is used as one of the input and the actual sequencing result is used as the output.

There is a group of *N’* (*N’* is a natural number) samples. In every sample, *s* (*s*>1) judges sort the objects *O*_1_,*O*_2_,…, *O*_*r*_. In the sample *N* (*N*≤*N’*), the sequencing judgment by the judge *i* (*i* = 1,2,…,*s*) can be expressed as a linear order $LI_{i}^{\left(N\right)}:O_{i1}^{\left(N\right)},O_{i2}^{\left(N\right)},\cdots O_{ir}^{\left(N\right)}$. The symbol $LI_{i}^{\left(N\right)}(O_{j})$, which represents the number of the objects behind *O*_*j*_ in *LI*_*i*_ (including itself), also represents the score of *O*_*j*_ in *LI*_*i*_.

**Fig 3:**
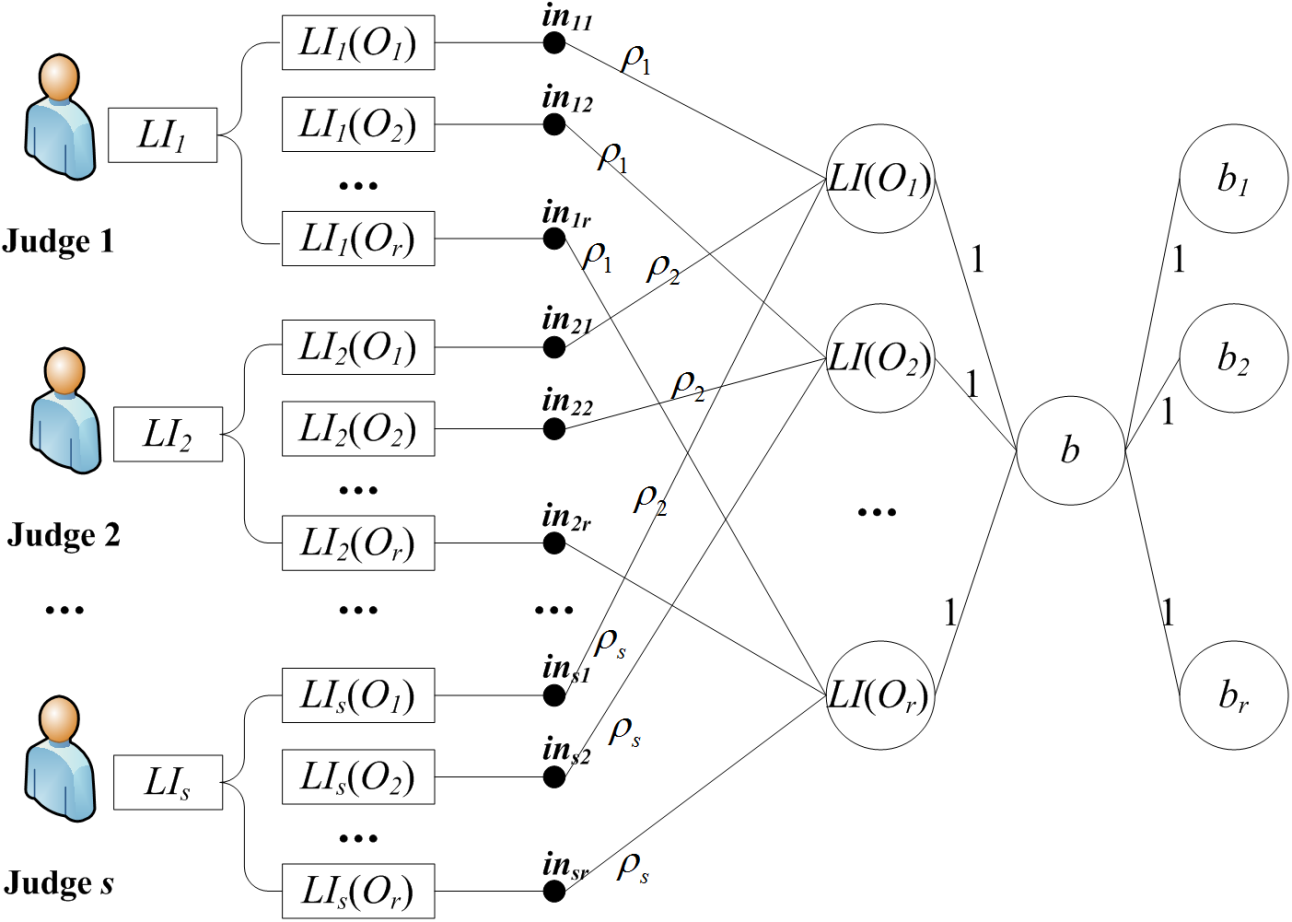
The structure of BP neural network for determining JID.

We assume that the JID vector is *ρ* = [*ρ*_1_,*ρ*_2_,⋯,*ρ*_*s*_] ($\sum_{i=1}^{s}\rho_{i}=1,0\leq \rho_{i}\leq 1$). $LI^{\left(N\right)}(O_{j})=\sum_{i=1}^{s}\rho_{i}\cdot LI_{i}^{\left(N\right)}(O_{j})$ is called the weighted Borda number of the object *O*_*j*_. Lastly, the objects *O*_1_,*O*_2_,…, *O*_*r*_ are sequenced by their weighted Borda numbers. The sequencing result of *s* judges is also a linear order $LI^{\left(N\right)}:O_{1}^{\left(N\right)},O_{2}^{\left(N\right)},\cdots ,O_{r}^{\left(N\right)}$. $LI^{*}:O_{1}^{*},O_{2}^{*},\cdots ,O_{r}^{*}$ is the actual sequencing linear order of the objects *O*_1_,*O*_2_,…, *O*_*r*_. So we can build the BP neural network model as shown in Fig 3.

In the input layer, the neuron number is *s×r* and the input matrix is $IN^{\left(N\right)}={\left(in_{ij}^{\left(N\right)}\right)}_{s\times r}$, where $in_{ij}^{\left(N\right)}$ is equal to $LI_{i}^{\left(N\right)}(O_{j})$.

In the hidden layer, the neuron number is *r* (equal to the object number). For the neuron *i*, its input is $y_{i}^{\left(N\right)}$ and output is $d_{i}^{\left(N\right)}$ (*i* = 1,2,…,*r*). The connection weight matrix between input layer and hidden layer is composed of *s* matrix blocks, and the matrix block *j* (*j* = 1,2,…,*s*) is:

$$\underset{j}{\Gamma^{\left(N\right)}}={\left(\underset{j}{\gamma_{ik}^{\left(N\right)}}\right)}_{r\times r}=\left[\begin{array}{cccc} \underset{j}{\gamma_{11}^{\left(N\right)}} & 0 & \cdots & 0\\ 0 & \underset{j}{\gamma_{22}^{\left(N\right)}} & \cdots & 0\\ \vdots & \vdots & & \vdots \\ 0 & 0 & \cdots & \underset{j}{\gamma_{rr}^{\left(N\right)}} \end{array}\right]$$ (7)

In the output layer, there is only one neuron. The connection weight matrix between hidden layer and output layer is (1,1,…,1)^*T*^.

The reasoning process can be described as follows. For the hidden layer node *j* (*j* = 1,2,…,*r*), its input is $y_{i}^{\left(N\right)}=\sum_{i=1}^{s}\rho_{i}^{\left(N\right)}in_{ij}^{\left(N\right)}=LI^{\left(N\right)}(O_{j})$ and its output is $d_{j}^{\left(N\right)}=f(y_{j}^{\left(N\right)}+\theta_{j})$ (the effect function is $f(y)=\frac{1}{1+e^{-y}}$). For the output layer node, its input is $d^{\left(N\right)}=(d_{1}^{\left(N\right)},d_{2}^{\left(N\right)},\cdots ,d_{r}^{\left(N\right)})$, and its output is $b^{\left(N\right)}=(b_{1}^{\left(N\right)},b_{2}^{\left(N\right)},\cdots ,b_{r}^{\left(N\right)})$ in which $b_{i}^{\left(N\right)}$ is the score of *O*_*i*_ in the linear order $LI^{\left(N\right)}:O_{1}^{\left(N\right)},O_{2}^{\left(N\right)},\cdots ,O_{r}^{\left(N\right)}$ sorted by $d_{1}^{\left(N\right)},d_{2}^{\left(N\right)},\cdots ,d_{r}^{\left(N\right)}$. Its effect is sorting *O*_1_,*O*_2_,…, *O*_*r*_ in order and giving their scores. For the error function, the error of the sample *N* is $Error^{\left(N\right)}=\sum_{j=1}^{r}{\left(b_{j}^{\left(N\right)}-b_{j}^{*}\right)}^{2}$ where $b_{j}^{\left(N\right)}$ is the score of *O*_*j*_ in $LI^{\left(N\right)}:O_{1}^{\left(N\right)},O_{2}^{\left(N\right)},\cdots ,O_{r}^{\left(N\right)}$ and $b_{j}^{*}$ is the score of *O*_*j*_ in $LI^{*}:O_{1}^{*},O_{2}^{*},\cdots ,O_{r}^{*}$.

**Fig 4:**
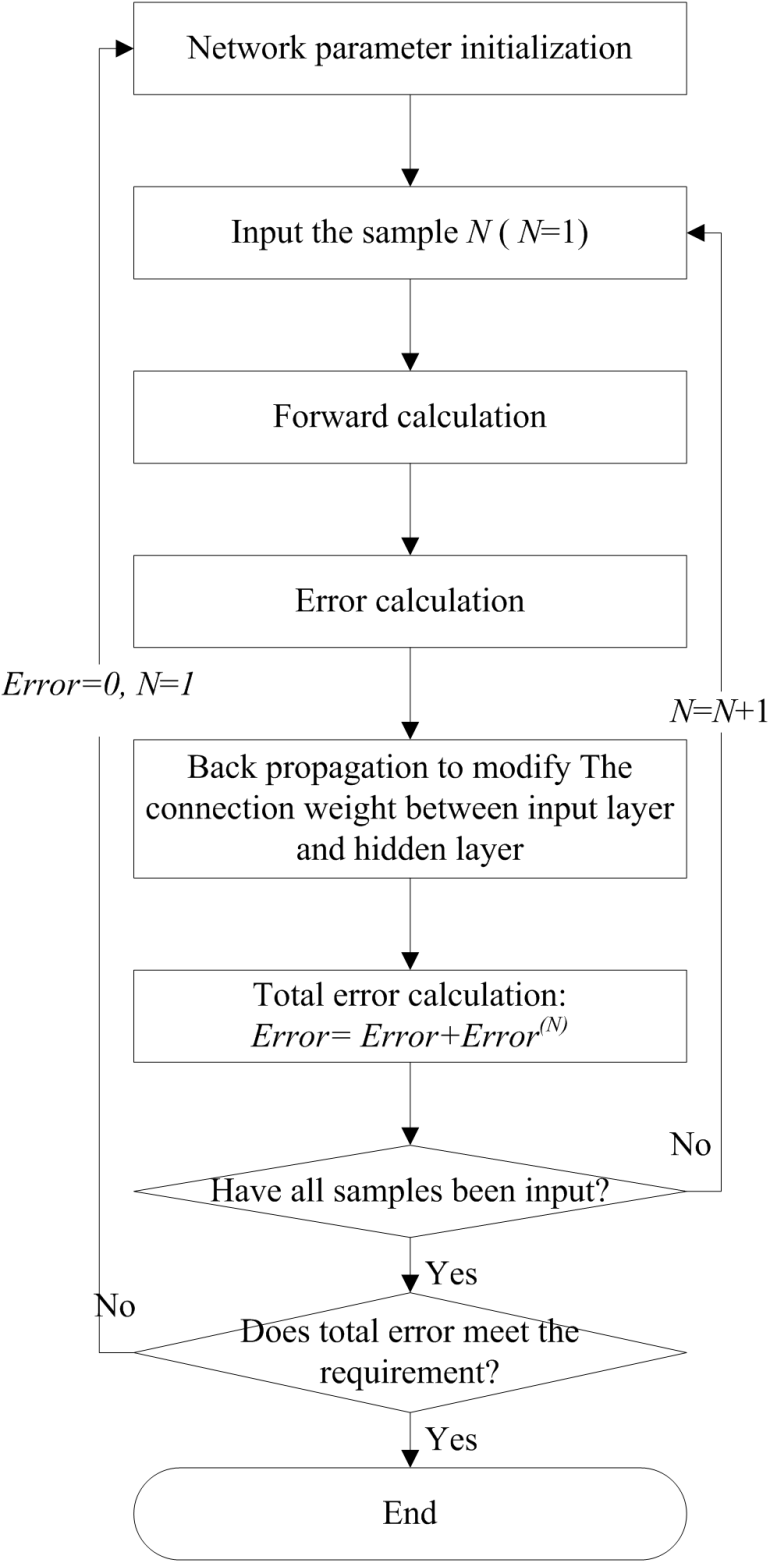
The learning process of BP neural network to determine JID.

The learning process is shown in Fig 4.

After the BP neural network has been trained successfully, we need normalize the connection weights between input layer and hidden layer to obtain the JID as follows:

For the connection weight matrix between input layer and hidden layer, calculate the diagonal elements’ average value of its every matrix block. To the matrix block *j* (*j* = 1,2,…,*s*), that is $\underset{j}{\gamma^{\left(N'\right)}}=\frac{1}{r}\sum_{i=1}^{r}\underset{j}{\gamma_{ii}^{\left(N'\right)}}$.

The average values $\underset{1}{\gamma^{\left(N'\right)}},\underset{2}{\gamma^{\left(N'\right)}},\cdots \underset{s}{\gamma^{\left(N'\right)}}$ are normalized and the importance degree of judge *j* is:

$$\rho_{j}=\underset{j}{\gamma^{\left(N'\right)}}/\sum_{k=1}^{s}\underset{k}{\gamma^{\left(N'\right)}}$$ (8)

So we can obtain that the JID vector is *ρ* = [*ρ*_1_,*ρ*_2_,⋯,*ρ*_*s*_].

#### Trapezoid Fuzzy Scale-Fuzzy AHP Considering the Judge Importance Degree

We use trapezoidal fuzzy number to express the subjective judgment opinion [51]. Based on the membership function of trapezoid fuzzy number, the natural number 1,2,…,*n* can be converted to trapezoidal fuzzy number:

$$\tilde{i}=\left\{ \begin{array}{l} (1,1,\frac{3}{2},2),\;i=1\\ (i-1,i-\frac{1}{2},i+\frac{1}{2},i+1),\;i=2,3,\cdots ,n-1\\ (n-1,n-\frac{1}{2},n,n),\;i=n \end{array}\right.$$ (9)

The comments of the 9-level scale are “extremely inferior”, “strongly inferior”, “significantly inferior”, “slightly inferior”, “equal”, “slightly superior”, “significantly superior”, “strongly superior” and “extremely superior”. Therefore, the 9-level scale 1, 2, …, 9 can be improved to the form of $\tilde{1}/\tilde{9},\tilde{2}/\tilde{8},\cdots ,\tilde{9}/\tilde{1}$. According to Eq 9, we can calculate that the trapezoidal fuzzy numbers $\tilde{1}/\tilde{9},\tilde{2}/\tilde{8},\cdots ,\tilde{9}/\tilde{1}$ equal to (1/9,1/9,3/17,1/4), (1/9,3/17,1/3,3/7), …, (4,17/3,9,9) in order.

Based on RS theory, it is assumed that *U* is the non-null limited target set namely discourse domain, and *T* is one target in *U*. All targets in *U* belong to *n* partitions *D*_*1*_,*D*_*2*_,*…*,*D*_*n*_ with the order of *D*_*1*_ *<D*_*2*_ *<…<D*_*n*_.

For *D*_*i*_ ∈ *R*, 1*≤i≤n*, its upper approximation set and lower approximation set are as follows:

$$\begin{array}{l} \bar{AS}(D_{i})=\left\{T\in \text{K}|\text{K}\subseteq U/R(T)\wedge K\geq D_{i}\right\}\\ \underline{AS}(D_{i})=\left\{T\in \text{K}|\text{K}\subseteq U/R(T)\wedge K\leq D_{i}\right\} \end{array}$$ (10)

where *U/R*(*T*) is the partition of uncertain relationship *R* on *U*.

If all targets in *U* are fuzzy number form, *D*_*i*_ can be expressed by its fuzzy number rough boundary interval as follows:

$$\begin{array}{l} RBI(D_{i})=[\underline{L}(D_{i}),\bar{L}(D_{i})]\\ \bar{L}(D_{i})=\sum \left[R(T)/\bar{N}(D_{i}\right)],T\in \bar{AS}(D_{i})\\ \underline{L}(D_{i})=\sum \left[R(T)/\underline{N}(D_{i}\right)],T\in \underline{AS}(D_{i}) \end{array}$$ (11)

where $\bar{N}(D_{i})$ and $\underline{N}(D_{i})$ are the numbers of targets in $\bar{AS}(D_{i})$ and $\underline{AS}(D_{i})$, respectively.

The subjective weight is determined by the trapezoid fuzzy scale-fuzzy AHP considering the judge importance degree (TFS-FAHP-JID) as follows:

**Step 1:** For *v*_2_, *v*_3_, *v*_4_ dominated by *v*_1_ as shown in Fig 1, let *O =* {*O*_1_,*O*_2_,…, *O*_*r*_} (here *r* = 3 and *O*_1_ = *v*_2_, *O*_2_ = *v*_3_, *O*_3_ = *v*_4_) and collect the opinions of *s* (*s*>1) judges.

The fuzzy reciprocal judgment matrix of judge *u* is ${\tilde{X}}^{\left(u\right)}={\left({{\tilde{x}}_{ij}}^{\left(u\right)}\right)}_{r\times r}$ where the trapezoidal fuzzy number ${{\tilde{x}}_{ij}}^{\left(u\right)}=(a_{ij}^{\left(u\right)},b_{ij}^{\left(u\right)},c_{ij}^{\left(u\right)},d_{ij}^{\left(u\right)})$ is the relative importance degree of *O*_*j*_ to *O*_*i*_ given by the judge *u*, *O*_*j*_,*O*_*i*_ ∈ *O*, *u* = 1,2,…,*s*. We use the form of $\tilde{1}/\tilde{9},\tilde{2}/\tilde{8},\cdots ,\tilde{9}/\tilde{1}$ to express ${{\tilde{x}}_{ij}}^{\left(u\right)}$.

**Step 2:** Do the consistency check on ${\tilde{X}}^{\left(1\right)},{\tilde{X}}^{\left(2\right)},\cdots ,{\tilde{X}}^{\left(s\right)}$, respectively. The simple form of ${\tilde{X}}^{\left(u\right)}$ is $X^{\left(u\right)}={\left(x_{ij}{}^{\left(u\right)}\right)}_{n\times n},x_{ij}{}^{\left(u\right)}\in \text{ker}({{\tilde{x}}_{ij}}^{\left(u\right)})$ where $\text{ker}({{\tilde{x}}_{ij}}^{\left(u\right)})$ represents the kern of ${{\tilde{x}}_{ij}}^{\left(u\right)}$. $hr_{i}{}^{\left(u\right)}=\sum_{j=1}^{r}x_{ij}{}^{\left(u\right)},hc_{j}{}^{\left(u\right)}=\sum_{i=1}^{r}x_{ij}{}^{\left(u\right)},ha^{\left(u\right)}=\sum_{i=1}^{r}\sum_{j=1}^{r}x_{ij}{}^{\left(u\right)}$, so $\phi^{\left(u\right)}=\underset{j}{\max}\{|\frac{1}{hc_{j}{}^{\left(u\right)}}-\frac{hr_{j}{}^{\left(u\right)}}{ha^{\left(u\right)}}|\}$. If *ϕ* ^(*u*)^ ≤ *ε* (*ε* is usually equal to 0.1), *X* ^(*u*)^ passes the consistent check, so ${\tilde{X}}^{\left(u\right)}$ is a consistent matrix. Otherwise some appropriate adjustments of ${\tilde{X}}^{\left(u\right)}$ are needed.

**Step 3:** Construct the group-decision matrix $\tilde{X}={\left({\tilde{x}}_{ij}\right)}_{\text{r}\times \text{r}}$ where ${\tilde{x}}_{ij}=\{{{\tilde{x}}_{ij}}^{\left(1\right)},{{\tilde{x}}_{ij}}^{\left(2\right)},\cdots ,{{\tilde{x}}_{ij}}^{\left(s\right)}\}$.

**Step 4:** Calculate the fuzzy number rough boundary interval of every element in $\tilde{X}$ and construct the fuzzy-number rough pair comparison matrix ${\tilde{X}}^{*}$.

The fuzzy number rough boundary interval of ${{\tilde{x}}_{ij}}^{\left(u\right)}$ can be calculated as follows:

$$RBI({{\tilde{x}}_{ij}}^{\left(u\right)})=\left[\underline{L}({{\tilde{x}}_{ij}}^{\left(u\right)}),\bar{L}({{\tilde{x}}_{ij}}^{\left(u\right)})\right]$$ (12)

According to the JID vector *ρ* = [*ρ*_1_,*ρ*_2_,⋯,*ρ*_*s*_] ($\sum_{i=1}^{s}\rho_{i}=1,0\leq \rho_{i}\leq 1$), the fuzzy number rough boundary interval of ${\tilde{X}}_{ij}$ is as follows:

$$\begin{array}{l} RBI({\tilde{X}}_{ij})=\left[\underline{L}({\tilde{X}}_{ij}),\bar{L}({\tilde{X}}_{ij})\right]\\ \underline{L}({\tilde{X}}_{ij})=\rho_{1}\underline{L}({{\tilde{x}}_{ij}}^{\left(1\right)})+\rho_{2}\underline{L}({{\tilde{x}}_{ij}}^{\left(2\right)})+\cdots +\rho_{s}\underline{L}({{\tilde{x}}_{ij}}^{\left(s\right)})\\ \bar{L}({\tilde{X}}_{ij})=\rho_{1}\bar{L}({{\tilde{x}}_{ij}}^{\left(1\right)})+\rho_{2}\bar{L}({{\tilde{x}}_{ij}}^{\left(2\right)})+\cdots +\rho_{s}\bar{L}({{\tilde{x}}_{ij}}^{\left(s\right)}) \end{array}$$ (13)

So the fuzzy-number rough pair comparison matrix ${\tilde{X}}^{*}$ is as follows:

$${\tilde{X}}^{*}=\left[\begin{array}{cccc} RBI({\tilde{X}}_{11}) & RBI({\tilde{X}}_{12}) & \cdots & RBI({\tilde{X}}_{1r})\\ RBI({\tilde{X}}_{21}) & RBI({\tilde{X}}_{22}) & \cdots & RBI({\tilde{X}}_{2r})\\ \vdots & \vdots & & \vdots \\ RBI({\tilde{X}}_{r1}) & RBI({\tilde{X}}_{r2}) & \cdots & RBI({\tilde{X}}_{rr}) \end{array}\right]$$ (14)

**Step 5:** Separate ${\tilde{X}}^{*}$ into the upper bound matrix ${\tilde{X}}^{*,+}$ and the lower bound matrix ${\tilde{X}}^{*,-}$ as follows:

$${\tilde{X}}^{*,+}=\left[\begin{array}{cccc} \bar{L}({\tilde{X}}_{11}) & \bar{L}({\tilde{X}}_{12}) & \cdots & \bar{L}({\tilde{X}}_{1r})\\ \bar{L}({\tilde{X}}_{21}) & \bar{L}({\tilde{X}}_{22}) & \cdots & \bar{L}({\tilde{X}}_{2r})\\ \vdots & \vdots & & \vdots \\ \bar{L}({\tilde{X}}_{r1}) & \bar{L}({\tilde{X}}_{r2}) & \cdots & \bar{L}({\tilde{X}}_{rr}) \end{array}\right],\;{\tilde{X}}^{*,-}=\left[\begin{array}{cccc} \underline{L}({\tilde{X}}_{11}) & \underline{L}({\tilde{X}}_{12}) & \cdots & \underline{L}({\tilde{X}}_{1r})\\ \underline{L}({\tilde{X}}_{21}) & \underline{L}({\tilde{X}}_{22}) & \cdots & \underline{L}({\tilde{X}}_{2r})\\ \vdots & \vdots & & \vdots \\ \underline{L}({\tilde{X}}_{r1}) & \underline{L}({\tilde{X}}_{r2}) & \cdots & \underline{L}({\tilde{X}}_{rr}) \end{array}\right]$$ (15)

**Step 6:** The gravity center of a trapezoidal fuzzy number can represent its characteristics to the maximum extent, so we map the upper and lower bound matrixes ${\tilde{X}}^{*,+}$ and ${\tilde{X}}^{*,-}$ into the gravity center form *X* ^*,−^ and *X* ^*,+^. Here, for the trapezoidal fuzzy number (*a*, *b*, *c*, *d*) where *a*≤ *b*≤ *c*≤ *d*, its gravity center is:

$$G=\frac{\left(d^{2}+d\cdot c+c^{2})+(b^{2}+b\cdot a+a^{2}\right)}{3(d+c-a-b)}$$ (16)

For *X* ^*,+^, the eigenvector corresponding to its eigenvalue is calculated as ${\left[\xi_{1}^{-},\xi_{2}^{-},\cdots ,\xi_{r}^{-}\right]}^{\text{T}}$. For *X* ^*,+^, the eigenvector corresponding to its eigenvalue is calculated as ${\left[\xi_{1}^{+},\xi_{2}^{+},\cdots ,\xi_{r}^{+}\right]}^{\text{T}}$. So the relative weight vector of *O*_1_,*O*_2_,…, *O*_*r*_ dominated by *v*_1_ is

$${\left[\eta_{1},\eta_{2},\cdots ,\eta_{r}\right]}^{\text{T}}$$ (17)

where $\eta_{i}=\frac{\tau }{2}(\frac{\xi_{i}^{-}}{\sum_{j=1}^{r}\xi_{j}^{-}}+\frac{\xi_{i}^{+}}{\sum_{j=1}^{r}\xi_{j}^{+}})$.

Here, because *r* = 3 and *O*_1_ = *v*_2_, *O*_2_ = *v*_3_, *O*_3_ = *v*_4_, [*η*_1_,*η*_2_,⋯,*η*_*r*_]^T^ is equal to ${\left[\delta_{2}^{1},\delta_{3}^{1},\delta_{4}^{1}\right]}^{\text{T}}$.

**Step 7:** Repeating the steps 1–6 top to down as in shown in Fig 1. For the *n*_*k*_ nodes *v*_*i*_,*v*_*i+1*_,…, $v_{i+n_{k}}$ on layer *k* to the node *v*_*j*_ on layer *k-*1, the relative weight vector is $\delta^{j}={\left[\delta_{i}^{j},\delta_{i+1}^{j},\cdots ,\delta_{i+n_{k}}^{j}\right]}^{\text{T}}$. In *δ* ^*j*^, the relative weights of nodes on layer *k* not dominated by *v*_*j*_ equal to zero.

**Step 8:** Do hierarchical general ordering.

For the overall target, the subjective weight vector of *n*_k-1_ nodes on layer *k*-1 is as follows:

$$\varphi^{\left(k-1\right)}={\left[\varphi_{1}^{\left(k-1\right)},\varphi_{2}^{\left(k-1\right)},\cdots ,\varphi_{n_{k-1}}^{\left(k-1\right)}\right]}^{\text{T}}$$ (18)

Relative to the node *v*_*j*_ on layer *k*-1, the weight vector of *n*_*k*_ nodes on layer *k* is as follows:

$$P_{j}^{\left(k\right)}={\left[p_{1,j}^{\left(k\right)},p_{2,j}^{\left(k\right)},\cdots ,p_{n_{k},j}^{\left(k\right)}\right]}^{\text{T}}$$ (19)

where the weights of nodes not dominated by the node *v*_*j*_ equal to zero.

Therefore, merge the matrices $P_{1}^{\left(k\right)},P_{2}^{\left(k\right)},\cdots ,P_{n_{k-1}}^{\left(k\right)}$ as follows:

$$P_{}^{\left(k\right)}=[P_{1}^{\left(k\right)},P_{2}^{\left(k\right)},\cdots ,P_{n_{k-1}}^{\left(k\right)}]$$ (20)

The weight vector of the nodes on layer *k* relative to the overall target is as follows::

$$\varphi^{\left(k\right)}=P^{\left(k\right)}\cdot \varphi^{\left(k-1\right)}=P^{\left(k\right)}\cdot P^{\left(k-1\right)}\cdots \varphi^{\left(2\right)}$$ (21)

where *φ* ^(2)^ is the weight vector of the nodes on layer 2 relative to the overall target.

Finally the subjective weight vector of nodes on layer *L* relative to the overall target is calculated out as *φ* ^(*L*)^.

### Balanced Weight Calculation Based on Multi-weight Contribution Balance Model

In comprehensive sequencing process, the sequencing result of evaluation objects depends on their weighted indicator values. Based on the combination of the objective weight and the subjective weight, the balanced weight is defined as follows:

$$\omega =\lambda_{1}\cdot \varphi^{\left(4\right)}+\lambda_{2}\cdot \chi$$ (22)

where *λ*_1_,*λ*_2_ (*λ*_1_,*λ*_2_ ≥ 0, *λ*_1_ + *λ*_2_ = 1) are the balanced coefficients of the subjective and objective weight, respectively.

For the evaluation object *i* (*i* = 1,2,…,*l*), the subjective weight contribution of the indicator *v*_*j*_ is $\lambda_{1}\varphi_{v_{j}}^{\left(4\right)}z_{i,v_{j}}$, while the objective weight contribution of the indicator *v*_*j*_ is $\lambda_{2}\chi_{v_{j}}z_{i,v_{j}}$ (*p+*5≤*j*≤*q+*7). For the evaluation object *i*, the multi-weight contribution deviation is:

$$\sum_{j=p+5}^{q+7}{\left(\lambda_{1}\varphi_{v_{j}}^{\left(4\right)}z_{i,v_{j}}-\lambda_{2}\chi_{v_{j}}z_{i,v_{j}}\right)}^{2}$$ (23)

Based on the equality relationship of different evaluation objects, the total multi-weight contribution deviation is:

$$\sum_{i=1}^{l}\sum_{j=p+5}^{q+7}{\left(\lambda_{1}\varphi_{v_{j}}^{\left(4\right)}z_{i,v_{j}}-\lambda_{2}\chi_{v_{j}}z_{i,v_{j}}\right)}^{2}$$ (24)

Therefore, the multi-weight contribution balance model is built as:

$$\min\sum_{i=1}^{l}\sum_{j=p+5}^{q+7}{\left(\lambda_{1}\varphi_{v_{j}}^{\left(4\right)}z_{i,v_{j}}-\lambda_{2}\chi_{v_{j}}z_{i,v_{j}}\right)}^{2},\;s.t.\;\lambda_{1},\lambda_{2}\geq 0,\lambda_{1}+\lambda_{2}=1$$ (25)

Here, *λ*_2_ can be expressed as 1−*λ*_1_, so we can obtain *λ*_1_,*λ*_2_ as follows:

$$\lambda_{1}=\frac{\sum_{i=1}^{l}\sum_{j=p+5}^{q+7}z_{i,v_{j}}{}^{2}\chi_{v_{j}}(\varphi_{v_{j}}^{\left(4\right)}+\chi_{v_{j}})}{\sum_{i=1}^{l}\sum_{j=p+5}^{q+7}z_{i,v_{j}}{}^{2}{\left(\varphi_{v_{j}}^{\left(4\right)}+\chi_{v_{j}}\right)}^{2}},\;\lambda_{2}=\frac{\sum_{i=1}^{l}\sum_{j=p+5}^{q+7}z_{i,v_{j}}{}^{2}\varphi_{v_{j}}^{\left(4\right)}(\varphi_{v_{j}}^{\left(4\right)}+\chi_{v_{j}})}{\sum_{i=1}^{l}\sum_{j=p+5}^{q+7}z_{i,v_{j}}{}^{2}{\left(\varphi_{v_{j}}^{\left(4\right)}+\chi_{v_{j}}\right)}^{2}}$$ (26)

### Improved TOPSIS Sequencing

In information theory, the difference degree between two *n*-dimensional uncertainty systems $\Psi_{A}=(\psi_{1}^{A},\psi_{2}^{A},\cdots ,\psi_{n}^{A})$ and $\Psi_{B}=(\psi_{1}^{B},\psi_{2}^{B},\cdots ,\psi_{n}^{B})$ can be measured by the relative entropy as follows:

$$RD_{A}^{B}=\sum_{i=1}^{n}(\psi_{i}^{A}\log\frac{\psi_{i}^{A}}{\psi_{i}^{B}}+(1-\psi_{i}^{A})\log\frac{1-\psi_{i}^{A}}{1-\psi_{i}^{B}})$$ (27)

where $\psi_{i}^{A},\psi_{i}^{B}\;(i=1,2,\ldots ,n)$ mean the appearance probability of the uncertain status ***i*** in the systems Ψ_*A*_,Ψ_*B*_ [52,53].

According to the standardized decision matrix *Z* and the balanced weight *ω*, we can obtain the weighted standardized decision matrix *H* = *ω* ⋅ *Z*. The relative entropy $RD_{A}^{B}$ has the characteristics as follows: $RD_{A}^{B}$≥0, and $RD_{A}^{B}$ = 0 only when Ψ_*A*_ = Ψ_*B*_ [52,53]. So we definite the followings:

For the evaluation object *k* (*k* = 1,2,…,*l*), the relative entropy distance (RED) from it to the positive ideal point *H* ^+^ is as follows:

$$RD_{k}^{+}=\sum_{j=1}^{m}(h_{i}^{+}\log\frac{h_{i}^{+}}{h_{k,j}}+(1-h_{i}^{+})\log\frac{1-h_{i}^{+}}{1-h_{k,j}})$$ (28)

And the relative entropy divergence from it to the negative ideal point *H* ^−^ is as follows:

$$RD_{k}^{-}=\sum_{j=1}^{m}(h_{i}^{-}\log\frac{h_{i}^{-}}{h_{k,j}}+(1-h_{i}^{-})\log\frac{1-h_{i}^{-}}{1-h_{k,j}})$$ (29)

where $H^{+}=[h_{1}^{+},h_{2}^{+},\cdots ,h_{m}^{+}],h_{i}^{+}=\max\{h_{1,i},h_{2,i},\cdots ,h_{l,i}\}$ and $H^{-}=[h_{1}^{-},h_{2}^{-},\cdots ,h_{m}^{-}],h_{i}^{-}=\min\{h_{1,i},h_{2,i},\cdots ,h_{l,i}\}$.

For the evaluation object *H* ^*k*^, the relative entropy distance closeness between it and the ideal points *H* ^+^,*H* ^−^ is $RC_{k}=RD_{k}^{-}/(RD_{k}^{+}+RD_{k}^{-})$ which has the characteristics as follows:

1. If *H* ^*k*^ = *H* ^*+*^, *RC*_*k*_ = 1.
2. If *H* ^*k*^ = *H* ^*-*^, *RC*_*k*_ = 0.
3. When $RD_{k}^{+}$ *→*0 (that is *H* ^*k*^ *≠H* ^*+*^ and *H* ^*k*^ *≠H* ^*-*^, *H* ^*k*^ *→H* ^*+*^), *RC*_*k*_ *→*1.

As can be seen, the relative entropy distance between the evaluation object and the ideal points fits well with the basic sequencing principles of TOPSIS, so the improved TOPSIS by replacing Euclidean distance with the relative entropy distance (TOPSIS-RED) is reasonable. We can calculate the relative entropy distance closeness between each evaluation object and the ideal points successively, and obtain the final task queue optimization result by sequencing them in the descending order.

## Case Study

In the assembly workshop of an engine manufacturing enterprise, the task attributes are as follows:

1. Primary production types: development production, small batch production, maintenance, repair and overhaul (by expert evaluation method shown in Table 1, the indicator values are 0.8000, 0.5600, 0.2600, 0.3600, 0.4800 in order).
2. Assembly lines: *PL*_1_, *PL*_2_, *PL*_3_. Their maximum line-loads are 5, 10, 8 in order, and the undertaken task numbers are 3, 7, 4 in order. The tasks of different production types are mixed executed.
3. Customer types: the companies CAC, SAC, XAC (by expert evaluation method shown in Table 1, the indicator values are 0.3600, 0.7400, 0.5600).
4. Assembly units: the rotor unit *ET*_*1*_ (composed of the turbine part *P*_*1*_), the combustion unit *ET*_*2*_ (composed of the combustor part *P*_*2*_) and the transmission unit *ET*_*3*_ (composed of the decelerator part *P*_*3*_ and the transmission casing *P*_*4*_).

There are five tasks *T*_1_, *T*_2_, *T*_3_, *T*_4_, *T*_5_ (corresponding to the assembly line *PL*_2_, *PL*_1_, *PL*_3_, *PL*_3_, *PL*_1_, respectively) which are shown in Table 2.

**Table 2:** The attributes of the five tasks *T*_1_, *T*_2_, *T*_3_, *T*_4_, *T*_5._

Task	*v*_12_	*v*_13_	*v*_14_	*v*_15_	*v*_16_	*v*_17_	*v*_18_	*v*_19_	*v*_20_	*v*_21_	*v*_22_
*T*_1_	100%	56%	89%	95%	especially emergency	emergency	development production	SAC	10	3	70%
*T*_2_	20%	31%	22%	12%	more emergency	emergency	overhaul	XAC	5	2	60%
*T*_3_	91%	19%	95%	11%	general	more emergency	small batch production	CAC	8	4	50%
*T*_4_	88%	43%	56%	90%	emergency	especially emergency	repair	SAC	8	4	50%
*T*_5_	46%	80%	13%	26%	more emergency	general	maintenance	CAC	5	2	60%

The indicator value decision matrix is as follows:

$$T=\left[\begin{array}{ccccccccccc} 1.0000 & 0.5600 & 0.8900 & 0.9500 & 0.8000 & 0.6000 & 0.8000 & 0.7400 & 1.0000 & 0.7500 & 0.7000\\ 0.2000 & 0.3100 & 0.2200 & 0.1200 & 0.4000 & 0.6000 & 0.4800 & 0.5600 & 0.5000 & 0.5000 & 0.6000\\ 0.9100 & 0.1900 & 0.9500 & 0.1100 & 0.2000 & 0.4000 & 0.5600 & 0.3600 & 0.8000 & 1.0000 & 0.5000\\ 0.8800 & 0.4300 & 0.5600 & 0.9000 & 0.6000 & 0.8000 & 0.3600 & 0.7400 & 0.8000 & 1.0000 & 0.5000\\ 0.4600 & 0.8000 & 0.1300 & 0.2600 & 0.4000 & 0.2000 & 0.2600 & 0.3600 & 0.5000 & 0.5000 & 0.6000 \end{array}\right]$$ (30)

(1) The objective weight

The standardized decision matrix is as follows:

$$Z=\left[\begin{array}{ccccccccccc} 1.0000 & 0.7000 & 0.9368 & 1.0000 & 1.0000 & 0.7500 & 1.0000 & 1.0000 & 1.0000 & 0.7500 & 0.7143\\ 0.2000 & 0.3875 & 0.2316 & 0.1263 & 0.5000 & 0.7500 & 0.6000 & 0.7568 & 0.5000 & 0.5000 & 0.8333\\ 0.9100 & 0.2375 & 1.0000 & 0.1158 & 0.2500 & 0.5000 & 0.7000 & 0.4865 & 0.8000 & 1.0000 & 1.0000\\ 0.8800 & 0.5375 & 0.5895 & 0.9474 & 0.7500 & 1.0000 & 0.4500 & 1.0000 & 0.8000 & 1.0000 & 1.0000\\ 0.4600 & 1.0000 & 0.1368 & 0.2737 & 0.5000 & 0.2500 & 0.3250 & 0.4865 & 0.5000 & 0.5000 & 0.8333 \end{array}\right]$$ (31)

**Fig 5:**
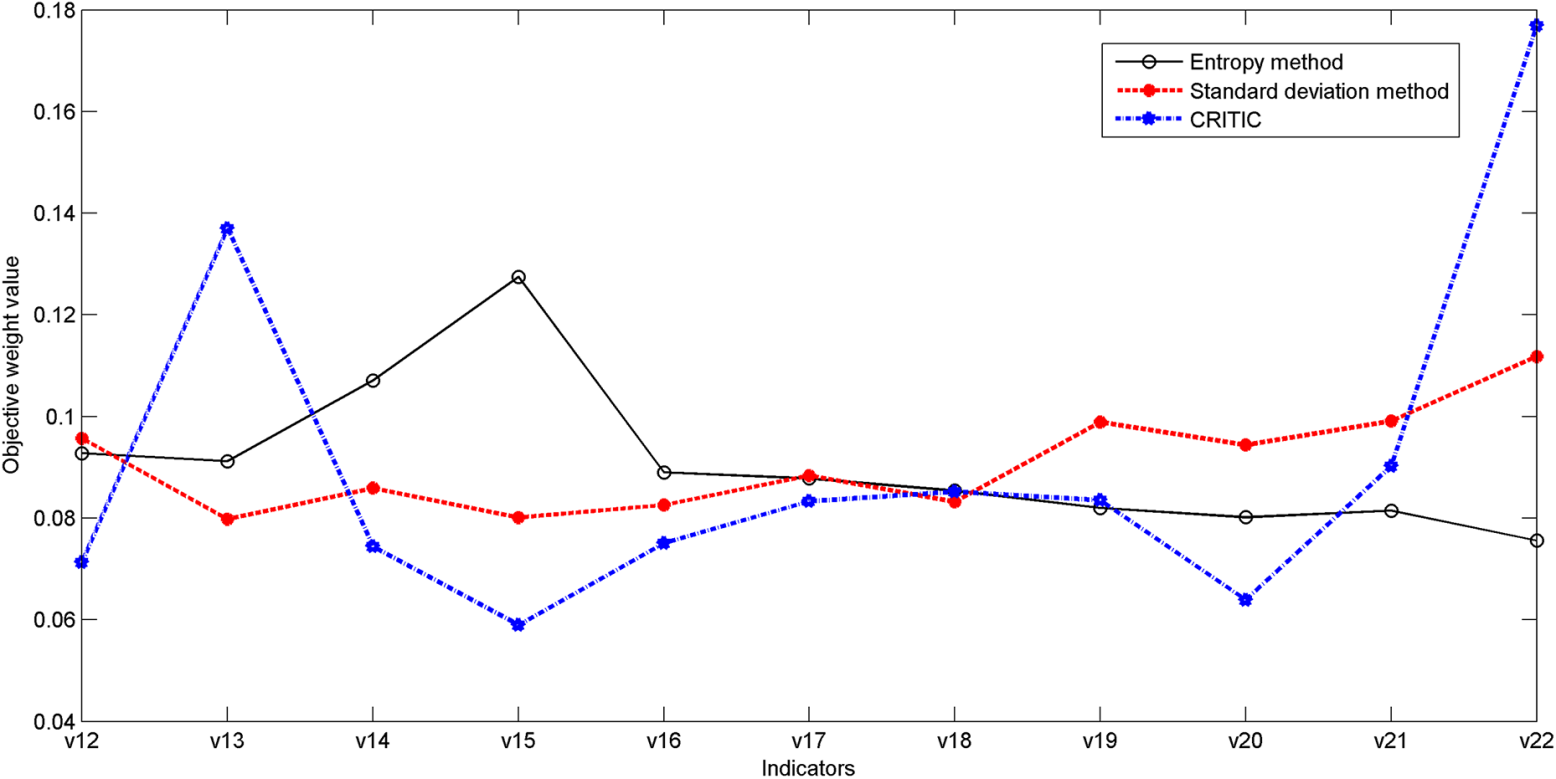
The comparison of objective weights using entropy method, standard deviation method, and CRITIC, respectively.

We can obtain three objective weight vectors by entropy method, standard deviation method and CRITIC shown in Table 3, respectively. Through the comparison shown in Fig 5, it is found that: The curve of the objective weight assignment determined by standard deviation method appears smooth. In general, the two curves of objective weight assignment determined by standard deviation method and entropy method are similar. The line of objective weight assignment determined by CRITIC is more complicated than the other two.

**Table 3:** Comparison of the objective weights determined by entropy method, standard deviation method and CRITIC.

Indicator	Entropy method		Standard deviation method		CRITIC		
	*EV*_*j*_	*χ*	*σ*_*j*_	*χ*	*σ*_*j*_	*R*_*j*_	*χ*
*v*_12_	0.7178	0.0928	0.6044	0.0957	0.6044	5.1520	0.0713
*v*_13_	0.7228	0.0912	0.5041	0.0798	0.5041	11.8650	0.1370
*v*_14_	0.6745	0.1071	0.5423	0.0859	0.5423	5.9896	0.0744
*v*_15_	0.6125	0.1275	0.5062	0.0801	0.5062	5.0917	0.0590
*v*_16_	0.7296	0.0890	0.5215	0.0826	0.5215	6.2877	0.0751
*v*_17_	0.7332	0.0878	0.5586	0.0884	0.5586	6.5125	0.0833
*v*_18_	0.7405	0.0854	0.5256	0.0832	0.5256	7.0750	0.0852
*v*_19_	0.7506	0.0820	0.6244	0.0989	0.6244	5.8377	0.0835
*v*_20_	0.7563	0.0802	0.5965	0.0944	0.5965	4.6755	0.0639
*v*_21_	0.7522	0.0815	0.6261	0.0991	0.6261	6.2889	0.0902
*v*_22_	0.7702	0.0756	0.7065	0.1118	0.7065	10.9308	0.1769

In conclusion, both entropy method and standard deviation method reduce the competition degree of different indicators, and make the objective weight assignment tends to equilibrium. So they cannot fully reflect the actual condition of the objective weight assignment. With the consideration of both the variability and the conflict, CRITIC can more fully reflect the competition information of the indicators than entropy method or standard deviation. The comparison and analysis are consistent with the method definitions in Section **Objective Weight**. So we choose CRITIC to determine the objective weight of the indicators. By CRITIC, the objective weight vector is as follows:

$$\chi ={\left[0.0713\;0.1370\;0.0744\;0.0590\;0.0751\;0.0833\;0.0852\;0.0835\;0.0639\;0.0902\;0.1769\right]}^{\text{T}}$$ (32)

(2) The subjective weight

It is assumed that there are three judges to sort four objects. There are six samples shown in Table 4. In the sample 1, the four objects are *O*_1_,*O*_*2*_,*O*_3_,*O*_4_ and their real order is *O*_2_,*O*_3_,*O*_4_,*O*_1_. In the sample 2, the four objects are *O*_5_,*O*_6_,*O*_7_,*O*_8_ and their real order is *O*_5_,*O*_6_,*O*_8_,*O*_7_. In the sample 3, the four objects are *O*_9_,*O*_10_,*O*_11_,*O*_12_ and their real order is *O*_10_,*O*_12_,*O*_11_,*O*_9_. In the sample 4, the four objects are *O*_13_,*O*_14_,*O*_15_,*O*_16_ and their real order is *O*_13_,*O*_16_,*O*_15_,*O*_14_. In the sample 5, the four objects are *O*_17_,*O*_18_,*O*_19_,*O*_20_ and their real order is *O*_20_,*O*_17_,*O*_18_,*O*_19_. In the sample 6, the four objects are *O*_21_,*O*_22_,*O*_23_,*O*_24_ and their real order is *O*_23_,*O*_22_,*O*_24_,*O*_21_.

**Table 4:** The six samples of the BP neural network model.

Sample No.	Input												Expected output			
	*in*_11_	*in*_12_	*in*_13_	*in*_14_	*in*_21_	*in*_22_	*in*_23_	*in*_24_	*in*_31_	*in*_32_	*in*_33_	*in*_34_	*b*_1_^*^	*b*_2_^*^	*b*_3_^*^	*b*_4_^*^
1	1	4	2	3	4	1	3	2	3	2	1	4	1	4	3	2
2	4	3	1	2	3	1	4	2	4	2	1	3	4	3	1	2
3	1	4	2	3	2	3	1	4	4	1	3	2	1	4	2	3
4	4	2	1	3	2	4	3	1	2	4	1	3	4	1	2	3
5	2	3	1	4	2	3	1	4	3	4	1	2	3	2	1	4
6	1	3	2	4	1	2	3	4	4	3	2	1	1	3	4	2

Due to space limitations, the detailed training process of BP neural network is omitted. The JID vector is calculated as follows:

ρ=[0.42850.33460.2369] (33)

For the nodes *v*_2_, *v*_3_, *v*_4_ dominated by the overall evaluation target layer node *v*_1_, trapezoid fuzzy scale-rough AHP is used to determine their relative subjective weights as follow:

**Step 1:** The fuzzy reciprocal judgment matrices of *v*_2_, *v*_3_, *v*_4_ by the three judges are as follows:

$${\tilde{X}}^{\left(1\right)}=\left[\begin{array}{ccc} \tilde{5}/\tilde{5} & \tilde{6}/\tilde{4} & \tilde{7}/\tilde{3}\\ \tilde{4}/\tilde{6} & \tilde{5}/\tilde{5} & \tilde{6}/\tilde{4}\\ \tilde{3}/\tilde{7} & \tilde{4}/\tilde{6} & \tilde{5}/\tilde{5} \end{array}\right],{\tilde{X}}^{\left(2\right)}=\left[\begin{array}{ccc} \tilde{5}/\tilde{5} & \tilde{7}/\tilde{3} & \tilde{8}/\tilde{2}\\ \tilde{3}/\tilde{7} & \tilde{5}/\tilde{5} & \tilde{6}/\tilde{4}\\ \tilde{2}/\tilde{8} & \tilde{4}/\tilde{6} & \tilde{5}/\tilde{5} \end{array}\right],{\tilde{X}}^{\left(3\right)}=\left[\begin{array}{ccc} \tilde{5}/\tilde{5} & \tilde{7}/\tilde{3} & \tilde{5}/\tilde{5}\\ \tilde{3}/\tilde{7} & \tilde{5}/\tilde{5} & \tilde{3}/\tilde{7}\\ \tilde{5}/\tilde{5} & \tilde{7}/\tilde{3} & \tilde{5}/\tilde{5} \end{array}\right]$$ (34)

**Step 2:** After calculation, ${\tilde{X}}^{\left(1\right)},{\tilde{X}}^{\left(2\right)},{\tilde{X}}^{\left(3\right)}$ all pass the consistency check.

**Step 3:** The group-decision matrix is constructed as follows:

$$\tilde{X}=\left[\begin{array}{ccc} \tilde{5}/\tilde{5},\tilde{5}/\tilde{5},\tilde{5}/\tilde{5} & \tilde{6}/\tilde{4},\tilde{7}/\tilde{3},\tilde{7}/\tilde{3} & \tilde{7}/\tilde{3},\tilde{8}/\tilde{2},\tilde{5}/\tilde{5}\\ \tilde{4}/\tilde{6},\tilde{3}/\tilde{7},\tilde{3}/\tilde{7} & \tilde{5}/\tilde{5},\tilde{5}/\tilde{5},\tilde{5}/\tilde{5} & \tilde{6}/\tilde{4},\tilde{6}/\tilde{4},\tilde{3}/\tilde{7}\\ \tilde{3}/\tilde{7},\tilde{2}/\tilde{8},\tilde{5}/\tilde{5} & \tilde{4}/\tilde{6},\tilde{4}/\tilde{6},\tilde{7}/\tilde{3} & \tilde{5}/\tilde{5},\tilde{5}/\tilde{5},\tilde{5}/\tilde{5} \end{array}\right]$$ (35)

**Step 4**: Calculate the fuzzy number rough boundary interval of every element in $\tilde{X}$.

We take ${\tilde{X}}_{12}=\{x_{12}^{\left(1\right)},x_{12}^{\left(2\right)},x_{12}^{\left(3\right)}\}=\{\tilde{6}/\tilde{4},\tilde{7}/\tilde{3},\tilde{7}/\tilde{3}\}$ for example. For the partition ‘$x_{12}^{\left(1\right)}=\tilde{6}/\tilde{4}$’ of ${\tilde{X}}_{12}$, its upper approximation set is $\left\{\tilde{6}/\tilde{4},\tilde{7}/\tilde{3},\tilde{7}/\tilde{3}\right\}$ and its lower approximation set is $\left\{\tilde{6}/\tilde{4}\right\}$. So the upper limit of $RBI(x_{12}^{\left(1\right)})$ is $\bar{L}(x_{12}^{\left(1\right)})=\frac{\tilde{6}/\tilde{4}+\tilde{7}/\tilde{3}+\tilde{7}/\tilde{3}}{3}$ = (0.7500,1.6455,2.6190,3.4444), and the lower limit of $RBI(x_{12}^{\left(1\right)})$ is $\underline{L}(x_{12}^{\left(1\right)})=\frac{\tilde{6}/\tilde{4}}{1}$ = (1.0000,1.2222,1.8571,2.3333). For the other two partitions ‘$x_{12}^{\left(2\right)}=\tilde{7}/\tilde{3}$’ and ‘$x_{12}^{\left(3\right)}=\tilde{7}/\tilde{3}$’, the upper and lower limits of $RBI(x_{12}^{\left(2\right)})$ and $RBI(x_{12}^{\left(3\right)})$ can be obtained by the same way. By Eq 13, $RBI({\tilde{X}}_{12})$ = [(0.8333,1.5044,2.3650,3.0740), (1.2500,1.7866,2.8730,3.8148)].

The fuzzy number rough boundary intervals of other elements in $\tilde{X}$ can be obtained similarly. The fuzzy-number rough pair comparison matrix ${\tilde{X}}^{*}$ can be constructed as follows:

$${\tilde{X}}^{*}=\left[\begin{array}{ccc} \left[\tilde{1},\tilde{1}\right] & RBI({\tilde{X}}_{12}) & RBI({\tilde{X}}_{13})\\ RBI({\tilde{X}}_{21}) & \left[\tilde{1},\tilde{1}\right] & RBI({\tilde{X}}_{23})\\ RBI({\tilde{X}}_{31}) & RBI({\tilde{X}}_{32}) & \left[\tilde{1},\tilde{1}\right] \end{array}\right]$$ (36)

**Step 5**: Separate ${\tilde{X}}^{*}$ into the upper bound matrix ${\tilde{X}}^{*,+}$ and the lower bound matrix ${\tilde{X}}^{*,-}$:

$${\tilde{X}}^{*,-}=\left[\begin{array}{ccc} \left(1,1,1,1\right) & \left(0.8333,1.5044,2.3650,3.0740\right) & \left(1.2870,1.4603,2.0741,2.7222\right)\\ \left(0.2698,0.3561,0.5695,0.7037\right) & \left(1,1,1,1\right) & \left(0.4167,0.5309,0.8315,1.0370\right)\\ \left(0.2485,0.3115,0.4644,0.5582\right) & \left(0.5476,0.6850,1.0606,1.3333\right) & \left(1,1,1,1\right) \end{array}\right]$$ (37)

$${\tilde{X}}^{*,+}=\left[\begin{array}{ccc} \left(1,1,1,1\right) & \left(1.2500,1.7866,2.8730,3.8148\right) & \left(1.9537,2.4603,4.4074,6.7222\right)\\ \left(0.3492,0.4473,0.6939,0.8519\right) & \left(1,1,1,1\right) & \left(0.9167,1.1235,1.7106,2.1481\right)\\ \left(0.6929,0.7233,0.7977,0.8439\right) & \left(0.7619,0.9451,1.5152,2.0000\right) & \left(1,1,1,1\right) \end{array}\right]$$ (38)

**Step 6**: By Eq 16, the upper and lower bound matrices ${\tilde{X}}^{*,+}$ and ${\tilde{X}}^{*,-}$ can be mapped into the gravity center matrices *X* ^*,−^ and *X* ^*,+^ as follows:

$$X^{*,-}=\left[\begin{array}{ccc} 1 & 1.9456 & 1.9018\\ 0.4761 & 1 & 0.7067\\ 0.3965 & 0.9106 & 1 \end{array}\right],X^{*,+}=\left[\begin{array}{ccc} 1 & 2.4448 & 3.9492\\ 0.5873 & 1 & 1.4815\\ 0.7649 & 1.3148 & 1 \end{array}\right]$$ (39)

The eigenvector corresponding to the eigenvalue of *X* ^*,−^ and *X* ^*,+^ is [-0.8394–0.3820–0.3865]^T^ and [-0.8431–0.3700–0.3901]^T^, respectively. So the relative weight vector of *v*_2_,*v*_3_,*v*_4_ dominated by *v*_1_ is [0.5240 0.2342 0.2419]^T^.

**Fig 6:**
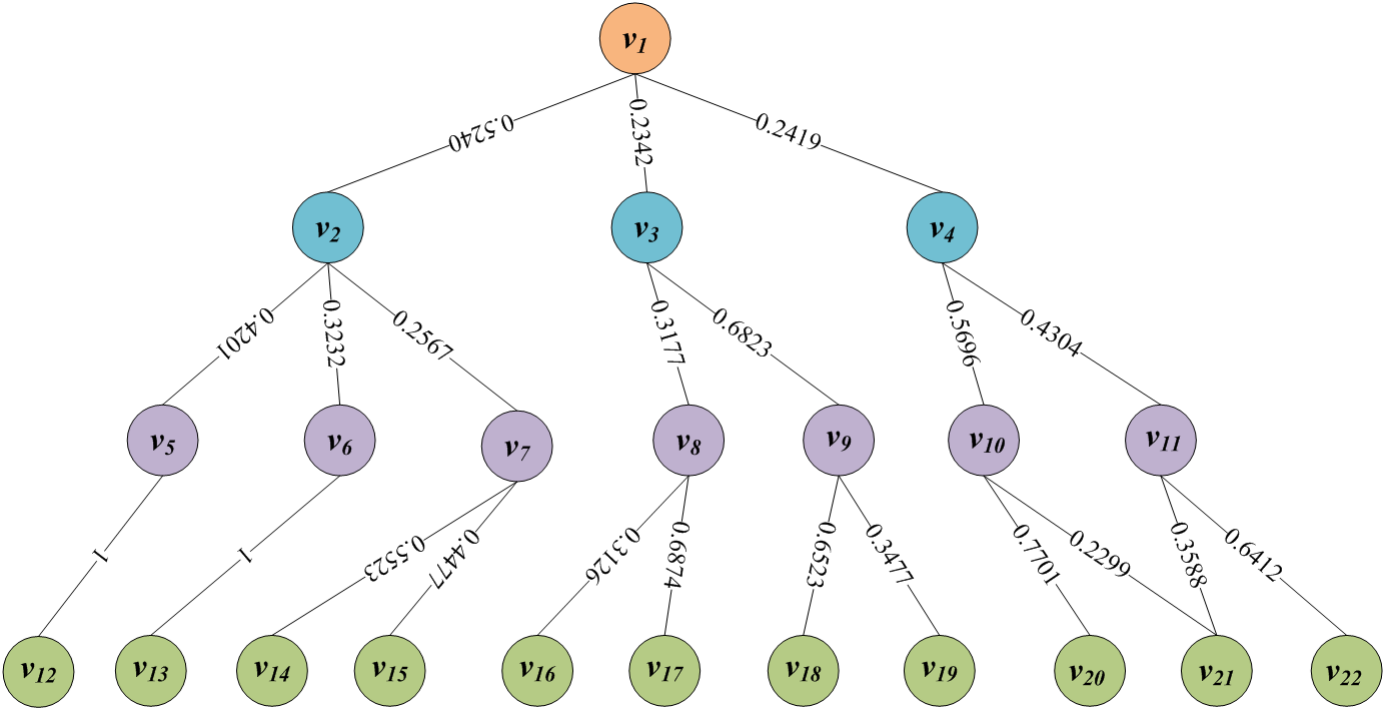
The relative subjective weights.

**Step 7:** Repeating the steps 1–6 from top to bottom as Fig 2, the relative subjective weights of all nodes to their upper node can be obtained as shown in Fig 6.

**Step 8:** After hierarchical general ordering, the subjective weight vector of the indicators is calculated out as follows:

$$\varphi^{\left(4\right)}={\left[0.2201\;0.1694\;0.0743\;0.0602\;0.0233\;0.0511\;0.1042\;0.0556\;0.1061\;0.0690\;0.0667\right]}^{\text{T}}$$ (40)

(3) The balanced weight

The standardized decision matrix is shown in Eq 31. We can obtain the balanced coefficients *λ*_1_ = 0.4894,*λ*_2_ = 0.5106 by Eq 26, so the balanced weight vector of the indicators is as follows:

$$\omega ={\left[0.1442\;0.1529\;0.0744\;0.0596\;0.0497\;0.0675\;0.0945\;0.0698\;0.0846\;0.0798\;0.1230\right]}^{\text{T}}$$ (41)

(4) Improved TOPSIS sequencing

According to the balanced weight vector *ω* in Eq 41 and the standardized decision matrix in Eq 31, the weighed standardized decision matrix is as follows:

$$H=\left[\begin{array}{ccccccccccc} 0.1442 & 0.1070 & 0.0697 & 0.0596 & 0.0497 & 0.0506 & 0.0945 & 0.0698 & 0.0846 & 0.0599 & 0.0879\\ 0.0309 & 0.0510 & 0.0241 & 0.0152 & 0.0279 & 0.0461 & 0.0609 & 0.0510 & 0.0408 & 0.0317 & 0.0408\\ 0.1405 & 0.0313 & 0.1040 & 0.0140 & 0.0140 & 0.0307 & 0.0812 & 0.0255 & 0.0653 & 0.0635 & 0.0490\\ 0.1359 & 0.0708 & 0.0613 & 0.1142 & 0.0419 & 0.0615 & 0.0406 & 0.0766 & 0.0653 & 0.0635 & 0.0490\\ 0.0710 & 0.1316 & 0.0142 & 0.0330 & 0.0279 & 0.0154 & 0.0203 & 0.0255 & 0.0408 & 0.0317 & 0.0408 \end{array}\right]$$ (42)

The positive ideal point is *H* ^*+*^ = (0.1442, 0.1529, 0.0744, 0.0596, 0.0497, 0.0675, 0.0945, 0.0698, 0.0846, 0.0798, 0.1230), and the negative ideal point is *H* ^*-*^ = (0.0288, 0.0363, 0.0102, 0.0069, 0.0124, 0.0169, 0.0307, 0.0340, 0.0423, 0.0399, 0.0879).

The task priority evaluation result of *T*_1_, *T*_2_, *T*_3_, *T*_4_, *T*_5_ is shown in Table 5, so the production task queue optimization result is *T*_1_> *T*_4_> *T*_3_> *T*_5_> *T*_2_.

**Table 5:** The evaluation results of *T*_1_, *T*_2_, *T*_3_, *T*_4_, *T*_5._

Task No.	The relative entropy distance from the task *k* to *H* ^*+*^	The relative entropy distance from the task *k* to *H* ^*-*^	The relative entropy distance closeness of the task *k*

*T*_*1*_	0.0229	0.2897	0.9269
*T*_*2*_	0.3687	0.0396	0.0970
*T*_*3*_	0.2575	0.1538	0.3739
*T*_*4*_	0.0666	0.2131	0.7618
*T*_*5*_	0.3076	0.0950	0.2359

## Analysis and Discussion

The objective and subjective weight vectors are shown in Eqs 32 and 40. On the one hand, the objective weight assignment is more average than the subjective weight assignment. The reason could be that the indicator value fluctuation of the five tasks is not very obvious. As shown in Table 2, a slightly larger indicator value difference exists in the component preparing of the parts *P*_1_,*P*_4_ (*v*_12_,*v*_15_) and the product emergency degree (*v*_16_), and it is reflected in the objective weight vector shown in Eq 32. On the other hand, the component preparing indicators (*v*_12_,*v*_13_,*v*_14_,*v*_15_) have larger subjective weights. In fact, the production task priority is seriously influenced by the component preparing, so the subjective weight assignment is consistent with the actual condition.

By TOPSIS-RED, only using the subjective weight the evaluation result of *T*_1_, *T*_2_, *T*_3_, *T*_4_, *T*_5_ is (0.9341 0.4705 0.2326 0.8614 0.1559), and only using the objective weight the evaluation result of *T*_1_, *T*_2_, *T*_3_, *T*_4_, *T*_5_ is (0.7066 0.0518 0.4161 0.7350 0.2937). So the sequencing results of only using the subjective weight and only using the objective weight are *T*_1_> *T*_4_> *T*_2_> *T*_3_> *T*_5_ and *T*_4_> *T*_1_> *T*_3_> *T*_5_> *T*_2_, respectively.

**Fig 7:**
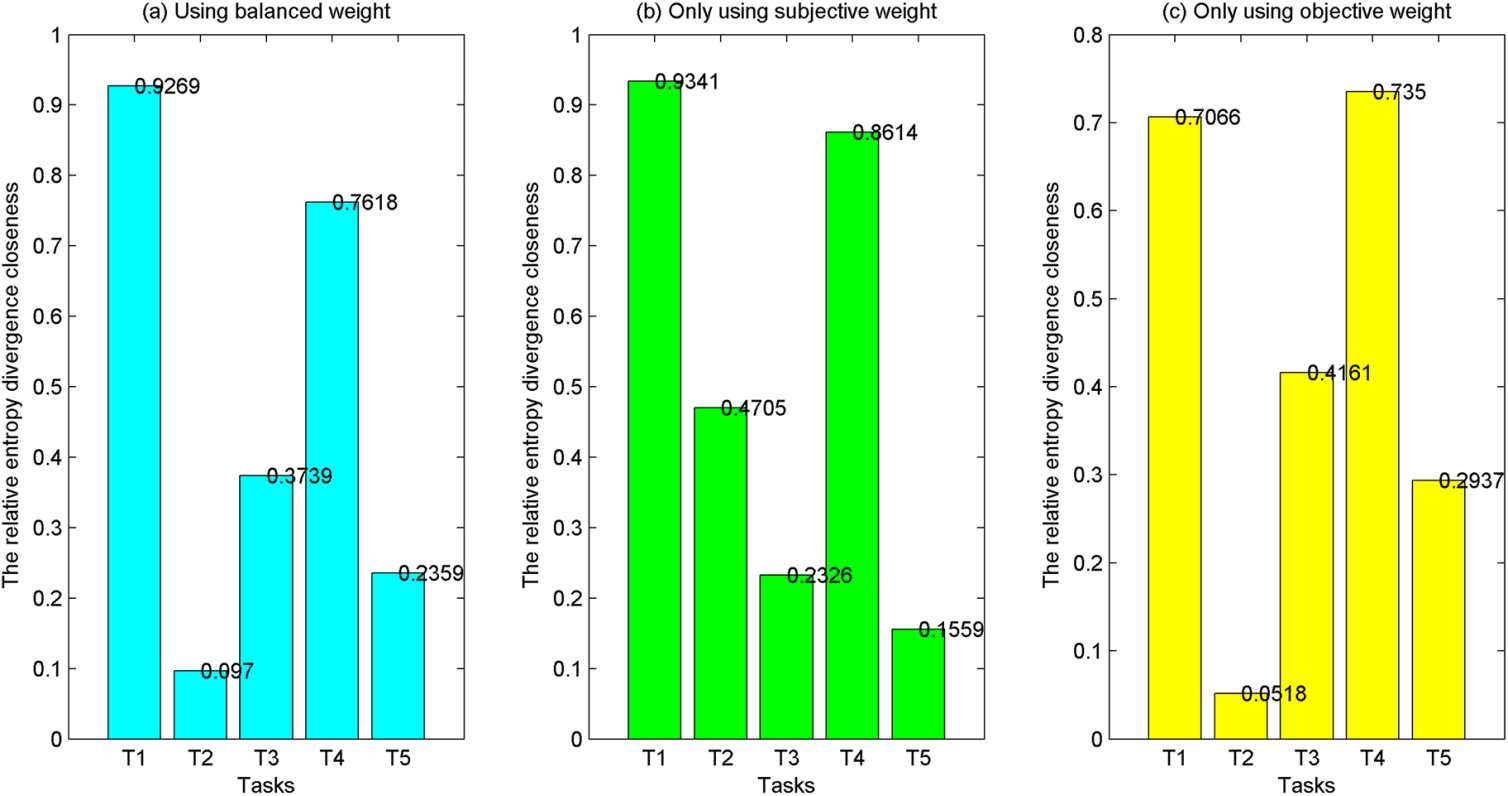
The sequencing results using the three weighting methods by TOPSIS-RED: (a) Using the balanced weight. (b) Only using the subjective weight. (c) Only using the objective weight.

As shown in Fig 7, the tasks *T*_1_, *T*_4_ have a higher priority than the tasks *T*_2_, *T*_3_, *T*_5_ in Fig 7(A), 7(B) and 7(C). However, several different sequencing details still exist among the three weighting methods. For example, *T*_1_ has a higher priority than *T*_4_ in Fig 7(A) and 7(B), but *T*_4_ has a higher priority than *T*_1_ in Fig 7(C). The sequencing result of *T*_2_, *T*_3_, *T*_5_ is *T*_3_> *T*_5_> *T*_2_ in Fig 7(A) and 7(C), but it is *T*_2_> *T*_3_> *T*_5_ in Fig 7(B). As can be seen, an information loss, which leads to the above different sequencing details, exists in the individual objective or subjective weight. The balanced weight, which integrates both the objective indicator value difference and the subjective judge preference, can reduce the information loss and neutralize the shortage of the individual weight.

**Fig 8:**
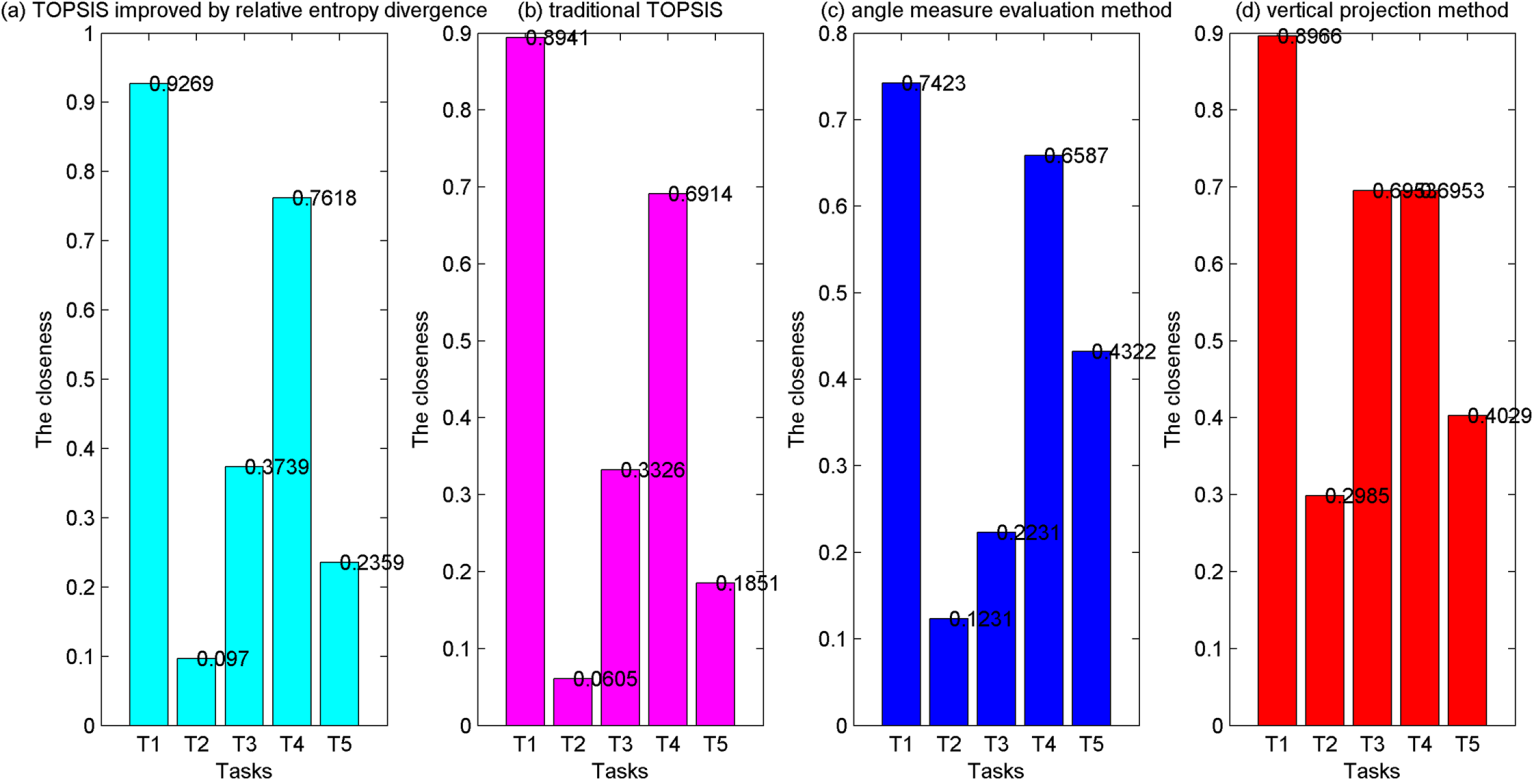
The sequencing results using the balanced weight by TOPSIS-RED and others: (a) TOPSIS-RED. (b) the traditional TOPSIS. (c) the improved TOPSIS by angle measure evaluation. (d) the improved TOPSIS by vertical projection method.

Using the balanced weight, the sequencing results of TOPSIS-RED, the traditional TOPSIS, the improved TOPSIS by angle measure evaluation and the improved TOPSIS by vertical projection method are shown in Fig 8. As can be seen, the sequencing result of TOPSIS-RED is as same as the result of the traditional TOPSIS, so the validity of TOPSIS-RED can be demonstrated. For the obvious insufficiency proved by many scholars [43,44], the traditional TOPSIS is not desirable.

As shown in Fig 8, the sequencing results, in which *T*_1_ has the highest priority and *T*_2_ has the lowest priority, are generally consistent.

By the improved TOPSIS by angle measure evaluation, the sequencing result of the evaluation objects *T*_3_ and *T*_5_ is *T*_3_ <*T*_5_ (Fig 8(C)), but by other three methods it is *T*_5_ <*T*_3_ (Fig 8(A), Fig 8(B) and Fig 8(D)). Only the angle closeness is considered, but the length difference is ignored. The evaluation objects *T*_3_ and *T*_5_ have the same angle closeness but different length, so we get the wrong sequencing result by the angle measure evaluation method.

By the improved TOPSIS by vertical projection method, the evaluation objects *T*_3_ and *T*_4_, which have the equal closeness to the ideal points, cannot be sequenced (Fig 8(D)).

Therefore, the improved TOPSIS by angle measure evaluation and the improved TOPSIS by vertical projection cannot meet the sequencing requirements in some special cases. It is consistent with the discussion in Section **Introduction**.

Based on the above analysis, the production task optimization method mainly has the advantages as follows:

1. The advantage of the balanced weight is proved as shown in Fig 6. To calculate the indicator weight, a combination balanced weighting method based on multi-weight contribution balance is proposed. Both the objective difference and the subjective judge preference are taken into consideration. The balanced weighting can reduce the information loss caused by the individual objective or subjective weight.
2. The advantage of TOPSIS-RED is proved as shown in Fig 6. To sequence the evaluation objects by their weighted indicator value, TOPSIS-RED is used to solve the problems of the traditional TOPSIS, the angle measure evaluation method and the vertical projection method. However, a large number of mathematical calculations exist in the proposed production task queue optimization method.

However, a large number of mathematical calculations exist in the proposed production task queue optimization method. The method may be very cumbersome and complex in the practical application. Although the trapezoidal fuzzy number is used, the subjective judge preference is still difficult to be expressed and quantified. In the future, more factors of the subjective judge thinking should be considered such as the judge preference history and experience etc.

## Conclusions

The production task queue has a great significance for manufacturing resource allocation and scheduling decision. The man-made qualitative method for production task queue optimization has a poor effect and makes the application difficult. A production task queue optimization method is proposed based on multi-attribute evaluation. The contribution and novelty are mainly as follows:

1. The hierarchical multi-attribute model is built based on the task attributes, and the definition and the quantization methods of the indicators are given.
2. The balanced weight, which integrates the objective weight and the subjective weight, is put forward based on the multi-weight contribution balance model.
  ➢ In the group decision, different judges have different experiences, abilities and employment positions. By the aid of the self-learning and training ability of BP neural network, a JID determination method is put forward.
  ➢ The exact number scale, which is used to express the subjective judgment in traditional AHP, cannot accurately reflect the ambiguity and uncertainty of the human mind. Using the trapezoidal fuzzy number to represent the ambiguity and uncertainty is more reasonable than the exact number scale, so TFS-RAHP-JID is put forward to calculate the subjective weight.
  ➢ Through the analysis and comparison of entropy method, standard deviation method and CRITIC, CRITIC is selected to calculate the objective weight.
  ➢ The balanced coefficients are introduced to achieve the multi-weight contribution balance.
3. TOPSIS-RED is put forward to sequence the evaluation objects by their weighted indicator value. The analysis and comparison proved that TOPSIS-RED is better than the traditional TOPSIS, the improved TOPSIS by angle measure evaluation and the improved TOPSIS by vertical projection. The case in an engine manufacturing enterprise’s assembly workshop illustrates the correctness and feasibility of the proposed method. The production task queue optimization result can provide the basis for manufacturing resource allocation and scheduling decision. Our future research will focus on the scheduling based on the production task queue optimization.
